# RNA-seq based identification and mutant validation of gene targets related to ethanol resistance in cyanobacterial *Synechocystis* sp. PCC 6803

**DOI:** 10.1186/1754-6834-5-89

**Published:** 2012-12-21

**Authors:** Jiangxin Wang, Lei Chen, Siqiang Huang, Jie Liu, Xiaoyue Ren, Xiaoxu Tian, Jianjun Qiao, Weiwen Zhang

**Affiliations:** 1 School of Chemical Engineering & Technology, Tianjin University, Tianjin, 300072, People's Republic of China; 2 Key Laboratory of Systems Bioengineering, Ministry of Education, Tianjin, 300072, People's Republic of China

**Keywords:** Ethanol, Tolerance, Transcriptomics, *Synechocystis*

## Abstract

**Background:**

Fermentation production of biofuel ethanol consumes agricultural crops, which will compete directly with the food supply. As an alternative, photosynthetic cyanobacteria have been proposed as microbial factories to produce ethanol directly from solar energy and CO_2_. However, the ethanol productivity from photoautotrophic cyanobacteria is still very low, mostly due to the low tolerance of cyanobacterial systems to ethanol stress.

**Results:**

To build a foundation necessary to engineer robust ethanol-producing cyanobacterial hosts, in this study we applied a quantitative transcriptomics approach with a next-generation sequencing technology, combined with quantitative reverse-transcript PCR (RT-PCR) analysis, to reveal the global metabolic responses to ethanol in model cyanobacterial *Synechocystis* sp. PCC 6803. The results showed that ethanol exposure induced genes involved in common stress responses, transporting and cell envelope modification. In addition, the cells can also utilize enhanced polyhydroxyalkanoates (PHA) accumulation and glyoxalase detoxication pathway as means against ethanol stress. The up-regulation of photosynthesis by ethanol was also further confirmed at transcriptional level. Finally, we used gene knockout strains to validate the potential target genes related to ethanol tolerance.

**Conclusion:**

RNA-Seq based global transcriptomic analysis provided a comprehensive view of cellular response to ethanol exposure. The analysis provided a list of gene targets for engineering ethanol tolerance in cyanobacterium *Synechocystis*.

## Background

Ethanol currently constitutes 99% of all biofuels in the United States. E-10 Unleaded, a blend of 10% ethanol and 90% ordinary gasoline, has been used in the U.S. for more than 25 years. Additionally, a blend of 85% ethanol and 15% ordinary gasoline (known as E-85) is rapidly growing in popularity [1]. The 3.4 billion gallons of ethanol blended into gasoline in 2004 amounted to about 2% of all gasoline sold by volume and 1.3% (2.5 x 1017 J) of its energy content [1]. Greater quantities of ethanol are expected to be used as a motor fuel in the future because of the federal policies, such as the “Twenty-in-Ten” program that proposes to cut gasoline consumption and greenhouse gas emissions from motor vehicles by 20 percent over the next 10 years. Large-scale ethanol production utilizes yeast or bacteria, such as *Saccharomyces cerevisiae* and *Zymomonas mobilis* to ferment sugar syrups [2]. The process has seen significant progress in recent years: inhibitor sensitivity, product tolerance, ethanol yield and specific ethanol productivity have been improved in modern industrial strains to the degree that up to 20% (*v*/*v*) of ethanol can be produced from starch-derived glucose [3]. However, since the large-scale ethanol fermentation consumes significant amount of agricultural crops, which competes directly with the world food supply, and its increased production has been blamed for the food price increases in recent years.

Photosynthetic cyanobacteria have recently attracted significant attention as a ‘microbial factory’ to produce biofuels and fine chemicals due to their capability to utilize solar energy and CO_2_ as sole energy and carbon sources, respectively [4]. By expressing a bacterial pyruvate decarboxylase (*pdc*) and alcohol dehydrogenase (*adh*) from the bacterium *Z. mobilis* in the cyanobacterium *Synechococcus* sp. PCC 7942, Deng and Coleman (1999) obtained a recombinant microorganism which can produce up to 230 mg/L ethanol directly from CO_2_ within 4 weeks of growth [5]. More recently, a genome-scale metabolic network model of *Synechocystis* sp. PCC 6803 was used to improve cyanobacterial ethanol production up to 690 mg/L in a week [6]. Although still at very low productivity, these works clearly demonstrated that photoautotrophic cyanobacteria could potentially be engineered for a direct conversion of solar energy and CO_2_ into biofuel products such as ethanol.

One of the key factors responsible for the low ethanol productivity is the low tolerance of photosynthetic systems to ethanol [7,8]. Ethanol can interfere with cell membrane’s ability to act as a barrier, and interrupt key cellular processes such as protein biosynthesis, energy transduction and transport [8]. Although ethanol tolerance mechanism and application of ethanol-tolerant strains for enhanced production have been reported in native-producing yeasts and bacteria, current knowledge on ethanol tolerance in cyanobacteria is not enough to guide a rational engineering of more robust cyanobacterial hosts. To address this issue, we previously applied a quantitative iTRAQ LC-MS/MS proteomics approach to determine the responses of model cyanobacterial *Synechocystis* sp. PCC 6803 to ethanol [9]. The analysis showed that the *Synechocystis* cells employed a combination of induced common stress response, modifications of cell membrane and envelope, and induction of multiple transporters and cell mobility-related proteins as major protection mechanisms against ethanol toxicity [9]. To further decipher responses at transcriptional level, in this study, we applied a quantitative transcriptomics approach with a next-generation sequencing technology, combined with quantitative reverse-transcript PCR (RT-PCR) analysis, to reveal the global metabolic responses to ethanol in *Synechocystis* sp. PCC 6803 [10]. We then compared the transcriptomics data with proteomic data obtained previously to further confirm the targets related to ethanol tolerance [9]. Finally, we constructed several knockout mutants of ethanol-induced genes to validate their potential application as targets for engineering ethanol tolerance. The RNA-seq transcriptomics analysis not only further confirmed the cellular responses revealed from previous proteomics analysis, but also showed that *Synechocystis* cells can also utilize enhanced PHA accumulation and glyoxalase detoxication pathway as means against ethanol stress. The study provided a list of gene targets for tolerance engineering in cyanobacterium *Synechocystis*.

## Results and discussion

### Ethanol effects on *Synechocystis* sp. PCC 6803

To make the transcriptomics data comparable with previous proteomics data, we used the identical sampling conditions for transcriptomics as our previous proteomics analysis [9]. As described before, the growth of *Synechocystis* sp. PCC 6803 supplemented with 0, 1.25, 1.50 and 2.00% ethanol was assessed to determine an appropriate ethanol concentration for proteomic studies. The results showed that the concentration of ethanol that caused a 50% growth decrease was found to be 1.50% (*v*/*v*) at 24 h (corresponding to middle-exponential phase), and was selected for the analysis in this study [9]. Cell morphology under ethanol-treated and control conditions was compared under microscope, and the results showed that visible aggregation of large number of cells was found after 24 h treatment even at a concentration of 1.50%, compared with the clearly individual cells in the control (data not shown). For transcriptomic analysis, two independent cultivations for both control (no ethanol) and 1.5% ethanol-treated experiments were conducted, and cells were collected by centrifugation (8,000 x *g* for 10 min at 4°C) at 24 h, 48 h and 72 h, resulting two biological replicates for each time point. The time points of sampling were corresponded to middle-exponential, exponential-stationary transition and stationary phases of the cell growth, respectively [9].

### Overview of transcriptomics analysis

**Figure 1:**
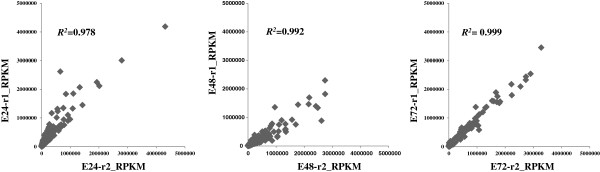
**Correlation of RNA-Seq data between biological replicates.** Normalized RPKM values from each sample were used. Correlation coefficients were indicated inside the plots.

A total of 112-million raw sequencing reads was obtained from the RNA-seq transcriptomics analysis of nine samples, with average reads of 12.5-million reads per sample. After a two-step data filtering process, first to eliminate reads with low-quality bases (such as multiple N) and reads shorter than 20 bp, and then to eliminate sequence reads mapped to non-coding RNA of *Synechocystis* sp. PCC 6803 [10], a total of 20.4-million qualified mRNA-based sequence reads were identified (Table 1). Except for the control sample at 24 h (Control-24 h) which has a genome mapping ratio of 57%, all other samples have mapping ratio larger than 60%, with the control sample at 72 h larger than 80%. Reproducibility between biological replicates of ethanol-treated samples at three time points was plotted (Figure 1), with correlation coefficient around 0.98-0.99, indicating the overall good quality of RNA sequencing. The sequence reads matched to all 3189 coding genes in *Synechocystis* sp. PCC 6803 genome (Additional file 1: Table S1), suggesting that the sequencing is deep enough to cover almost all species of transcripts in the cells. Abundance of the qualified mRNA-based raw sequence reads ranged from 1 to 341,135 for control samples, and from 1 to 154,326 for ethanol-treated samples, respectively, representing an expression dynamic range of 10^5^, which is higher than 10^3-4^ of typical microarray-based analyses [11,12]. Using Reads Per Kilobase of Gene per Million Mapped Reads (RPKM) as an index of the normalized transcript abundance [13], we identified the top expressed genes under the control and ethanol-treated conditions through the growth time course (Table 2). The top 50 expressed genes were found involved mostly in energy metabolism, including genes coding photosynthesis-related phycocyanin alpha subunit, phycocyanin beta subunit and photosystem I subunit XI and genes coding multiple subunits of ATP synthase, followed by genes encoding proteins synthesis such as multiple 50S ribosomal proteins and elongation factor, and genes involved in CO_2_ fixation such as ribulose bisphosphate carboxylase genes, consistent well with previous analysis on highly expressed genes in *Synechocystis*[14,15]. Interestingly, we also found several genes encoding hypothetical proteins (*ssl0483*, *slr0144*, *slr1470* and *slr0373*) were also among the top expressed genes, suggesting possible important physiological functions they may be responsible for. Although the exact function is still unknown, *slr0144* has been suggested to encode a PSII-associated protein [16], and *slr0373* forms an operon with *slr0374* which has been found responsive to various environmental stresses [17].

**Table 1:** Statistics of RNA-Seq transcriptomics analysis

**Sample ID**	**Raw sequences reads**	**Qualified mRNA reads**	**Genome-mapped Reads**	**Mapping ratio**
Control-24 h	18,859,596	5,552,572	3,177,168	57.22%
Control-48 h	10,589,107	2,331,738	1,784,966	76.55%
Control-72 h	11,249,884	3,026,360	2,455,571	81.14%
Ethanol-24 h-r1	12,277,943	3,211,398	1,985,207	61.82%
Ethanol-24 h-r2	13,736,430	3,755,422	2,323,029	61.86%
Ethanol-48 h-r1	13,384,367	4,041,104	2,453,989	60.73%
Ethanol-48 h-r2	10,306,514	2,934,288	1,772,957	60.42%
Ethanol-72 h-r1	11,883,271	3,623,746	2,363,916	65.23%
Ethanol-72 h-r2	9,814,306	3,160,336	2,083,580	65.93%

**Table 2:** Top 50 expressed genes based on normalized expression level (RPKM values)

**Gene ID**	**Control-24h**	**Control-48h**	**Control-72h**	**Ethanol-24h-r1**	**Ethanol-24h-r2**	**Ethanol-48h-r1**	**Ethanol-48h-r2**	**Ethanol-72h-r1**	**Ethanol-72h-r2**	**Discription**	**Cellular role**
***sll1744***	7.42E+05	8.96E+05	9.57E+05	6.14E+05	6.66E+05	6.65E+05	3.97E+05	7.24E+05	6.49E+05	50S ribosomal protein L1	Protein synthesis
***sll1745***	3.42E+06	3.74E+06	3.97E+06	2.80E+06	3.01E+06	2.74E+06	1.82E+06	2.90E+06	2.54E+06	50S ribosomal protein L10	Protein synthesis
***sll1743***	3.56E+05	5.73E+05	6.30E+05	3.00E+05	3.86E+05	4.17E+05	2.23E+05	4.90E+05	4.11E+05	50S ribosomal protein L11	Protein synthesis
***sll1746***	6.76E+05	5.38E+05	7.26E+05	1.66E+05	2.49E+05	2.90E+05	1.78E+05	2.78E+05	2.58E+05	50S ribosomal protein L12	Protein synthesis
***sll0767***	2.07E+06	9.55E+05	2.40E+06	9.92E+05	9.52E+05	7.76E+05	6.25E+05	7.37E+05	6.30E+05	50S ribosomal protein L20	Protein synthesis
***sll1801***	3.47E+05	5.18E+05	7.09E+05	1.62E+05	2.61E+05	4.74E+05	2.32E+05	5.43E+05	5.32E+05	50S ribosomal protein L23	Protein synthesis
***sll1807***	4.12E+05	6.41E+05	9.77E+05	1.72E+05	3.12E+05	5.68E+05	2.51E+05	6.14E+05	5.94E+05	50S ribosomal protein L24	Protein synthesis
***ssl1426***	5.89E+06	2.35E+06	5.49E+06	4.31E+06	4.18E+06	2.74E+06	2.30E+06	2.22E+06	2.17E+06	50S ribosomal protein L35	Protein synthesis
***slr2067***	1.00E+06	2.39E+06	4.47E+06	3.52E+05	1.16E+06	2.61E+06	8.80E+05	3.28E+06	3.45E+06	Allophycocyanin alpha subunit	Energy metabolism
***slr1986***	8.68E+05	1.50E+06	2.98E+06	2.53E+05	6.79E+05	1.35E+06	4.90E+05	1.72E+06	1.75E+06	Allophycocyanin beta subunit	Energy metabolism
***slr1198***	1.72E+06	1.22E+06	1.77E+06	1.08E+06	1.33E+06	1.10E+06	7.41E+05	1.08E+06	1.12E+06	Ant ioxidant protein	Unclassified
***sll1322***	2.11E+05	6.36E+05	8.15E+05	1.31E+05	4.50E+05	6.64E+05	3.23E+05	9.71E+05	7.65E+05	ATP synthase A chain of CF(0)	Energy metabolism
***ssl2615***	4.95E+05	1.22E+06	2.06E+06	2.69E+05	7.74E+05	1.33E+06	5.88E+05	1.82E+06	1.57E+06	ATP synthase C chain of CF(0)	Energy metabolism
***sll1323***	2.28E+05	5.71E+05	8.68E+05	1.17E+05	3.22E+05	6.10E+05	2.82E+05	8.06E+05	6.33E+05	ATP synthase subunit b' of CF(0)	Energy metabolism
***sll1099***	4.39E+05	9.53E+05	1.51E+06	1.94E+05	4.07E+05	7.88E+05	3.36E+05	9.11E+05	8.63E+05	Elongation factor Tu	Protein synthesis
***ssl0020***	2.52E+06	6.33E+05	7.00E+05	6.84E+05	7.65E+05	8.17E+05	7.10E+05	8.75E+05	7.41E+05	Ferredoxin I, essential for growth	Energy metabolism
***sll0018***	1.97E+05	7.10E+05	1.21E+06	1.36E+05	2.37E+05	7.34E+05	3.61E+05	1.04E+06	1.08E+06	Fructose-bisphosphate aldolase, class II	Unclassified
***ssl0483***	1.48E+06	5.07E+05	6.64E+05	6.22E+05	7.23E+05	6.12E+05	5.26E+05	6.88E+05	6.09E+05	Hypothetical protein	No Data
***slr0144***	8.77E+05	5.27E+05	5.68E+05	3.89E+05	4.55E+05	3.04E+05	2.59E+05	2.49E+05	2.15E+05	Hypothetical protein	Hypothetical proteins
***slr1470***	6.22E+05	6.04E+05	8.56E+05	4.92E+05	6.35E+05	6.70E+05	4.85E+05	7.30E+05	6.91E+05	Hypothetical protein	Hypothetical proteins
***slr0373***	4.60E+05	5.51E+05	1.65E+06	1.51E+05	3.63E+05	7.25E+05	2.74E+05	9.89E+05	8.96E+05	Hypothetical protein	No Data
***slr0749***	9.08E+05	6.46E+05	1.19E+06	7.58E+05	1.34E+06	7.50E+05	4.19E+05	4.46E+05	4.20E+05	Light-independent protochlorophyllide reductase ironprotein subunit ChlL	Cofactor biosynthesis
***slr0749***	9.08E+05	6.46E+05	1.19E+06	7.58E+05	1.34E+06	7.50E+05	4.19E+05	4.46E+05	4.20E+05	Light-independent protochlorophyllide reductase iron protein subunit ChlL	Energy metabolism
***sll1342***	3.21E+05	5.41E+05	7.43E+05	2.32E+05	3.29E+05	4.17E+05	2.38E+05	4.80E+05	5.42E+05	NAD(P)-dependent glyceraldehyde-3-phosphate dehydrogenase	Energy metabolism
***slr1834***	1.46E+06	1.55E+07	3.92E+07	1.23E+06	5.28E+06	1.28E+07	3.37E+06	1.50E+07	1.66E+07	P700 apoprotein subunit Ia	Energy metabolism
***slr1835***	1.21E+06	5.21E+06	1.16E+07	6.51E+05	2.62E+06	5.15E+06	1.74E+06	5.93E+06	5.79E+06	P700 apoprotein subunit Ib	Energy metabolism
***sll0819***	1.20E+06	6.37E+05	6.10E+05	3.32E+05	2.99E+05	3.72E+05	2.59E+05	4.10E+05	3.91E+05	Photosystem I reaction center subunit III precursor	Energy metabolism
***slr0737***	1.85E+06	1.15E+06	2.11E+06	9.16E+05	1.10E+06	1.20E+06	8.97E+05	1.34E+06	1.38E+06	Photosystem I subunit II	Energy metabolism
***smr0004***	4.02E+06	3.59E+06	5.24E+06	1.43E+06	1.45E+06	1.78E+06	1.44E+06	1.60E+06	1.58E+06	Photosystem I subunit VIII	Energy metabolism
***slr1655***	1.08E+07	5.24E+06	5.95E+06	8.83E+06	8.55E+06	7.79E+06	5.87E+06	7.50E+06	6.75E+06	Photosystem I subunit XI	Energy metabolism
***slr0906***	2.96E+05	5.56E+05	4.91E+05	1.78E+05	4.12E+05	5.30E+05	2.60E+05	7.12E+05	6.19E+05	Photosystem II core light harvest ing protein	Energy metabolism
***sll0851***	2.98E+05	1.21E+06	2.33E+06	1.71E+05	4.95E+05	1.05E+06	3.40E+05	9.30E+05	9.78E+05	Photosystem II CP43 protein	Energy metabolism
***slr1311***	2.37E+06	1.75E+06	2.79E+06	8.49E+05	1.83E+06	2.41E+06	1.39E+06	2.73E+06	2.44E+06	Photosystem II D1 protein	Energy metabolism
***sll1867***	1.22E+06	1.06E+06	1.62E+06	5.25E+05	1.01E+06	1.33E+06	7.68E+05	1.54E+06	1.59E+06	Photosystem II D1 protein	Energy metabolism
***sll0849***	1.31E+05	1.09E+06	1.59E+06	9.21E+04	3.93E+05	1.05E+06	3.10E+05	1.21E+06	1.20E+06	Photosystem II react ion center D2 protein	Energy metabolism
***slr0335***	4.12E+05	1.38E+06	2.12E+06	2.41E+05	6.30E+05	1.09E+06	5.13E+05	1.30E+06	1.35E+06	Phycobilisome core-membrane linker polypept ide	Energy metabolism
***sll1580***	3.09E+06	2.89E+06	2.91E+06	1.93E+06	2.24E+06	2.18E+06	1.69E+06	2.23E+06	1.79E+06	Phycobilisome rod linker polypept ide	Energy metabolism
***sll1579***	2.30E+06	2.95E+06	3.31E+06	1.33E+06	2.07E+06	2.16E+06	1.46E+06	2.54E+06	2.09E+06	Phycobilisome rod linker polypept ide	Unclassified
***slr2051***	1.44E+06	6.37E+05	7.37E+05	4.63E+05	5.46E+05	6.26E+05	5.26E+05	6.77E+05	6.51E+05	Phycobilisome rod-core linker polypeptide	Energy metabolism
***ssl3093***	2.10E+06	1.29E+06	1.36E+06	7.71E+05	9.40E+05	8.59E+05	7.79E+05	1.04E+06	7.32E+05	Phycobilisome small rod linker polypeptide	Unclassified
***sll1578***	2.59E+07	5.16E+07	8.62E+07	1.43E+07	2.65E+07	4.10E+07	2.17E+07	5.20E+07	5.40E+07	Phycocyanin alpha subunit	Energy metabolism
***sll1577***	2.49E+07	5.74E+07	8.88E+07	1.88E+07	3.58E+07	5.52E+07	2.70E+07	7.35E+07	7.72E+07	Phycocyanin beta subunit	Energy metabolism
***sll1694***	5.95E+06	8.44E+05	1.14E+06	2.01E+06	2.12E+06	9.58E+05	1.35E+06	1.07E+06	5.81E+05	Pilin polypept ide PilA1	Cell envelope
***sll0199***	8.92E+05	6.80E+05	9.96E+05	2.02E+05	5.59E+05	9.24E+05	4.93E+05	8.49E+05	7.72E+05	Plastocyanin	Unclassified
***slr0011***	1.94E+06	3.06E+06	6.38E+06	1.11E+06	1.85E+06	2.48E+06	1.33E+06	2.73E+06	2.32E+06	Possible Rubisco chaperonin	Hypothetical proteins
***slr1841***	1.24E+06	2.09E+06	3.27E+06	5.53E+05	1.31E+06	1.70E+06	7.51E+05	1.67E+06	1.89E+06	Probable porin; major outer membrane protein	Unclassified
***slr0009***	4.48E+05	1.44E+06	2.53E+06	2.81E+05	7.69E+05	1.33E+06	5.61E+05	1.70E+06	1.55E+06	Ribulose bisphosphate carboxylase large subunit	Energy metabolism
***slr0012***	5.54E+05	8.40E+05	2.10E+06	1.58E+05	2.52E+05	4.06E+05	2.72E+05	4.43E+05	3.79E+05	Ribulose bisphosphate carboxylase small subunit	Energy metabolism
***sll1338***	9.93E+05	2.56E+06	4.45E+06	5.64E+05	1.23E+06	1.57E+06	9.14E+05	1.79E+06	1.52E+06	Unknown protein	No Data
***sll1951***	7.54E+05	8.37E+05	1.25E+06	3.14E+05	6.19E+05	7.53E+05	3.27E+05	7.44E+05	8.16E+05	Unknown protein	Unclassified

**Figure 2:**
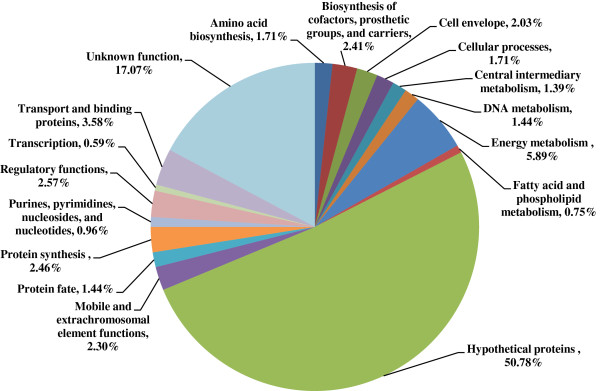
Pie chart of down-regulated genes by functional categories.

Using a cutoff of 1.5-fold change in both biological replicates, we determined that 1874 and 274 genes were down- and up-regulated by ethanol, respectively. For the down-regulated genes, 1343, 596 and 830 genes were down-regulated at 24, 48 and 72 h, respectively. Among them 167 genes were down-regulated in all three time points (Additional file 2: Table S2). Analysis of the functional category of the down-regulated genes was shown in Figure 2. The results showed that the most affected functional categories were “hypothetical proteins” and “unknown function”, representing a total of more than 68% of all the down-regulated genes, consistent with the fact that nearly half of the *Synechocystis* genome is still annotated as hypothetical up to now [10,18]. Other most affected functional categories included “Energy metabolism”, “Protein synthesis” and “Regulatory functions”. Down–regulation of the central metabolism is consistent with the overall slower growth upon ethanol stress [9]. For the up-regulated genes, 29, 114 and 161 genes were up-regulated at 24 h, 48 h and 72 h, respectively, among which 3 genes were up-regulated in all three time points (Table 3 and Additional file 3: Table S3). More genes up-regulated at late growth phases suggested that cells needed time to adjust their metabolism and initiate resistance responses.

**Table 3:** Genes induced by ethanol exposure *

**Gene ID**	**Description**	**Ratio-Ethanol-24h-r1 *vs. *Control-24h**	**Ratio-Ethanol-24h-r2 *vs. *Control-24h**	**Ratio-Ethanol-48h-r1 *vs. *Control-48h**	**Ethanol-48h-r2 *vs. *Control-48h**	**Ethanol-72h-r1 *vs. *Control-72h**	**Ratio-Ethanol-72h-r1 *vs. *Control-72h**
*sll0034*	putative carboxypeptidase			**2.69**	**1.50**		
*sll0250*	pantothenate metabolism flavoprotein					**3.00**	**1.91**
*sll0289*	septum site-determining protein MinD					**4.56**	**3.89**
*sll0300*	riboflavin synthase alpha chain					**4.33**	**3.33**
*sll0330*	sepiapterine reductase					**7.09**	**29.09**
*sll0368*	uracil phosphoribosyltransferase					**2.33**	**2.00**
*sll0374*	urea transport system ATP-binding protein			**2.80**	**3.60**		
*sll0384*	Cations and iron carrying protein			**2.31**	**1.70**		
*sll0450*	cytochrome b subunit of nitric oxide reductase	**1.62**	**4.26**				
*sll0536*	probable potassium channel protein			**2.00**	**1.93**		
*sll0540*	phosphate-binding protein PstS homolog					**1.57**	**1.79**
*sll0613*	Holliday junction DNA helicase RuvB			**1.73**	**1.91**		
*sll0621*	putative c-type cytochrome biogenesis protein CcdA					**2.22**	**2.00**
*sll0629*	alternative photosystem I reaction center subunit X			**1.94**	**1.72**		
*sll0671*	probable cation transporter	**4.00**	**4.00**				
*sll0686*	probable cytochrome c-type biogenesis protein					**3.00**	**3.25**
*sll0687*	RNA polymerase ECF-type (group 3) sigma factor			**3.00**	**6.00**		
*sll0759*	ABC transporter ATP-binding protein					**2.13**	**2.09**
*sll0759*	ABC transporter ATP-binding protein					**2.13**	**2.09**
*sll0792*	Zinc-responsive repressor ZiaR			**3.43**	**3.00**		
*sll0856*	RNA polymerase ECF-type (group 3) sigma-E factor					**1.89**	**1.61**
*sll1041*	similar to sulfate transport ATP-binding protein CysA			**2.63**	**1.63**		
*sll1051*	phycocyanin alpha-subunit phycocyanobilin lyase					**8.00**	**13.00**
*sll1051*	phycocyanin alpha-subunit phycocyanobilin lyase					**8.00**	**13.00**
*sll1170*	unknown protein					**2.25**	**1.50**
*sll1223*	diaphorase subunit of the bidirectional hydrogenase	**1.55**	**2.18**				
*sll1226*	hydrogenase subunit of the bidirectional hydrogenase					**1.55**	**1.89**
*sll1316*	cytochrome b6-f complex iron-sulfur subunit					**1.69**	**1.56**
*sll1330*	two-component system response regulator OmpR subfamily			**2.47**	**2.33**		
*sll1370*	mannose-1-phosphate guanylyltransferase					**3.25**	**1.88**
*sll1423*	global nitrogen regulator			**1.57**	**1.55**		
*sll1428*	probable sodium-dependent transporter	**7.00**	**6.00**			**5.00**	**2.00**
*sll1440*	pyridoxamine 5'-phosphate oxidase					**3.00**	**1.50**
*sll1471*	phycobilisome rod-core linker polypeptide	**3.90**	**2.77**	**3.56**	**3.07**		
*sll1473*	a part of phytochrome-like sensor histidine kinase gene			**1.59**	**1.79**		
*sll1483*	periplasmic protein					**2.00**	**8.40**
*sll1545*	glutathione S-transferase			**2.00**	**1.60**		
*sll1612*	folylpolyglutamate synthase	**2.00**	**2.67**				
*sll1679*	periplasmic protease HhoA	**3.35**	**2.42**				
*sll1682*	alanine dehydrogenase					**2.91**	**2.27**
*sll1723*	probable glycosyltransferase					**1.53**	**4.80**
*sll1724*	probable glycosyltransferase					**2.11**	**4.89**
*sll1994*	porphobilinogen synthase (5-aminolevulinate dehydratase)					**1.97**	**1.83**
*sll1998*	putative transposase [ISY100d: 1623697–1624643]					**1.67**	**4.00**
*slr0018*	fumarase					**2.27**	**1.67**
*slr0051*	periplasmic beta-type carbonic anhydrase			**1.55**	**1.60**		
*slr0070*	methionyl-tRNA formyltransferase					**1.62**	**1.69**
*slr0086*	similar to DnaK protein					**1.50**	**2.50**
*slr0089*	gamma-tocopherol methyltransferase					**4.60**	**3.80**
*slr0091*	aldehyde dehydrogenase					**7.00**	**11.00**
*slr0242*	bacterioferritin comigratory protein homolog			**1.58**	**1.65**		
*slr0328*	low molecular weight phosphotyrosine protein phosphatase					**2.75**	**2.00**
*slr0381*	lactoylglutathione lyase					**5.14**	**5.00**
*slr0502*	cobalamin synthesis protein cobW homolog	**1.75**	**1.88**			**1.83**	**1.67**
*slr0574*	cytochrome P450					**2.34**	**2.41**
*slr0585*	argininosuccinate synthetase					**2.32**	**2.42**
*slr0618*	cobyric acid synthase			**1.69**	**1.56**		
*slr0678*	biopolymer transport ExbD like protein					**2.19**	**2.26**
*slr0721*	malic enzyme					**1.67**	**2.29**
*slr0724*	HtaR suppressor protein homolog					**2.00**	**1.50**
*slr0741*	transcriptional regulator					**2.13**	**2.63**
*slr0758*	circadian clock protein KaiC homolog			**2.16**	**1.52**		
*slr0819*	apolipoprotein N-acyltransferase					**2.17**	**1.67**
*slr0898*	ferredoxin--nitrite reductase			**1.65**	**1.53**		
*slr0903*	molybdopterin (MPT) converting factor, subunit 2	**3.00**	**2.50**				
*slr0940*	zeta-carotene desaturase			**1.87**	**2.00**	**2.03**	**1.70**
*slr0942*	alcohol dehydrogenase [NADP+]			**1.76**	**1.58**		
*slr0946*	arsenate reductase					**4.50**	**3.50**
*slr0947*	response regulator for energy transfer from phycobilisomes to photosystems					**2.56**	**1.74**
*slr0949*	Integral membrane protein of the ABC-type Nat permease NatD					**14.00**	**7.00**
*slr1093*	2-amino-4-hydroxy-6-hydroxymethyldihydropteridine pyrophosphokinase			**1.73**	**1.94**		
*slr1109*	similar to ankyrin					**2.09**	**1.53**
*slr1120*	type 4 prepilin-like proteins leader peptide processing enzyme	**8.00**	**3.00**				
*slr1185*	cytochrome b6-f complex alternative iron-sulfur subunit					**8.00**	**5.00**
*slr1197*	SMF protein					**1.73**	**1.53**
*slr1204*	protease			**9.52**	**1.96**	**2.45**	**6.65**
*slr1205*	similar to chlorobenzene dioxygenase, ferredoxin component					**4.00**	**4.00**
*slr1225*	serine/threonine kinase					**2.70**	**2.50**
*slr1291*	NADH dehydrogenase subunit 4					**2.44**	**2.44**
*slr1300*	similar to 2-octaprenyl-6-methoxyphenol hydroxylase					**1.59**	**1.59**
*slr1350*	acyl-lipid desaturase					**1.72**	**1.57**
*slr1379*	quinol oxidase subunit I	**1.56**	**1.70**				
*slr1418*	dihydroorotate dehydrogenase					**2.50**	**2.63**
*slr1452*	sulfate transport system substrate-binding protein	**1.83**	**2.00**				
*slr1596*	a protein in the cytoplasmic membrane					**2.31**	**2.08**
*slr1626*	dihydroneopterin aldolase			**1.80**	**3.30**		
*slr1805*	two-component sensor histidine kinase					**1.60**	**1.98**
*slr1828*	ferredoxin, petF-like protein			**2.00**	**2.33**	**5.50**	**9.00**
*slr1848*	histidinol dehydrogenase					**2.15**	**2.06**
*slr1848*	histidinol dehydrogenase					**2.15**	**2.06**
*slr1853*	carboxymuconolactone decarboxylase	**1.80**	**1.70**	**2.71**	**1.59**	**2.56**	**2.55**
*slr1854*	unknown protein			**2.10**	**1.84**	**1.88**	**1.89**
*slr1874*	D-alanine--D-alanine ligase			**1.84**	**1.88**		
*slr1877*	2-hydroxyhepta-2,4-diene-1,7-dioate isomerase			**2.00**	**1.92**		
*slr1884*	tryptophanyl-tRNA synthetase					**2.09**	**1.72**
*slr1910*	probable N-acetylmuramoyl-L-alanine amidase					**1.71**	**2.00**
*slr1933*	dTDP-4-dehydrorhamnose 3,5-epimerase					**3.50**	**5.50**
*slr1938*	putative translation initiation factor EIF-2b subunit 1			**2.45**	**2.00**		
*slr1962*	probable extracellular solute-binding protein			**1.79**	**1.50**		
*slr1993*	PHA-specific beta-ketothiolase					**1.93**	**2.00**
*slr1994*	PHA-specific acetoacetyl-CoA reductase	**6.00**	**9.00**	**5.00**	**2.00**	**2.57**	**2.29**
*slr2033*	membrane-associated rubredoxin	**1.85**	**3.38**			**2.23**	**1.56**
*slr2114*	perosamine synthetase					**7.00**	**6.00**
*slr2131*	RND multidrug efflux transporter					**3.45**	**3.44**
*slr2143*	L-cysteine/cystine lyase	**1.67**	**2.56**				
*smr0003*	cytochrome b6-f complex subunit PetM					**2.40**	**3.40**
*smr0009*	photosystem II PsbN protein			**1.56**	**1.78**		
*ssl0563*	photosystem I subunit VII					**1.76**	**1.56**
*ssl0707*	nitrogen regulatory protein P-II			**2.00**	**1.64**		
*ssl2153*	probable ribose phosphate isomerase B			**4.50**	**2.50**		
*ssl2296*	pterin-4a-carbinolamine dehydratase			**1.75**	**1.86**		
*ssl2542*	high light-inducible polypeptide HliA, CAB/ELIP/HLIP superfamily					**4.00**	**5.00**
*ssl3580*	putative hydrogenase expression/formation protein HypC			**3.15**	**2.05**		
*ssr1176*	putative transposase			**1.50**	**1.83**		
*ssr1480*	putative RNA-binding protein			**2.42**	**1.92**		

* Induced genes encoding hypothetical proteins are provided in Additional file 3: Table S3.

### Correlation with quantitative RT-PCR analysis

**Figure 3:**
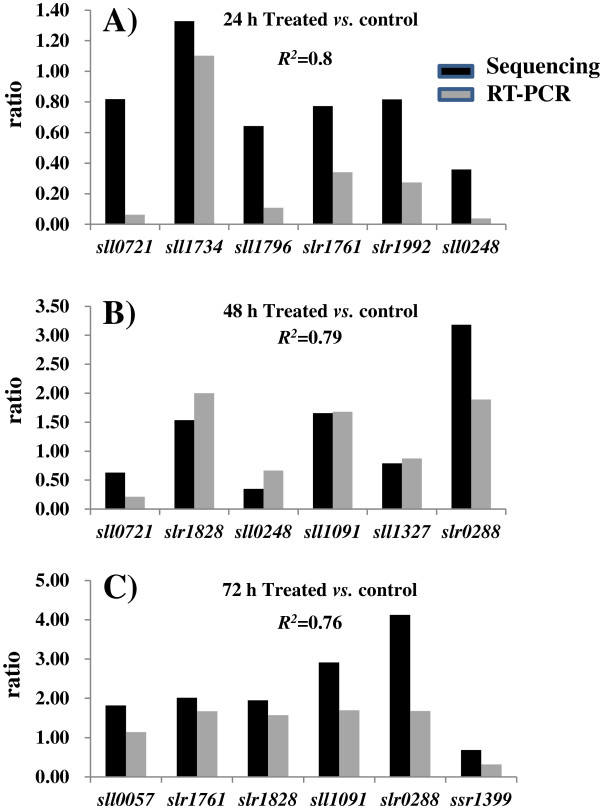
**Comparison of ratios derived from RNA-seq with that from RT-PCR analysis for selective genes. A**) 24 h; **B**) 48 h; and **C**) 72 h. Correlation coefficients were indicated inside the tables.

Based on their expression level and regulation patterns by ethanol, a subset of 12 genes was selected for quantitative RT-PCR validation. Among them, six genes were down-regulated (*i.e. sll0721*, *sll1796*, *slr1992*, *sll0248*, *sll1327*, *ssr1399*) and six genes were up-regulated (*i.e. sll1734*, *slr1761*, *slr1828*, *sll1091*, *slr0288*, *sll0057*) by ethanol, respectively according to the RNA-seq transcriptomics data. Under control condition, their expression levels varied from the normalized RPKM values 2529.6 for *sll0248* (encoding a flavodoxin) to 421749.3 for *ssr1399* (encoding ribosomal protein S18) (Additional file 4: Table S4). RT-PCR analysis was performed for the genes between the treated sample and control for all three time points (*i.e.* 24, 48 and 72 h). The results showed obvious positive correlation can be detected between qRT-PCR and RNA-Seq transcriptomics data (with correlation coefficient of 0.75-0.8) (Figure 3), suggesting a good quality of RNA-seq data.

### Cells utilize multiple approaches to cope with ethanol stress

Our previous proteomic analysis found that the *Synechocystis* cells employed a combination of induced common stress response, modifications of cell membrane and envelope, and induction of multiple transporters and cell mobility-related proteins as protection mechanisms against ethanol toxicity [9]. At transcriptional level, a very similar response was also observed [9]. First, we found that common stress responses were induced: one gene encoding a heat-shock DnaK homolog (*slr0086*) was induced at 72 h. Multiple genes involved in resistance against reactive oxygen species (ROS), such as *slr2033* encoding a membrane-associated rubredoxin, *slr1109* encoding ankyrin homolog [19], *sll1545* encoding glutathione S-transferase [20], *slr0242* encoding a bacterioferritin comigratory protein [21] and *slr1379* encoding quinol oxidase subunit I [22] were up-regulated. In addition, consistent with findings from proteomic analysis, we found circadian rhythms of *Synechocystis* sp. PCC 6803 was also regulated by ethanol. It was reported that cyanobacterial circadian rhythms are controlled by a cluster of three genes, *kaiA*, *kaiB*, and *kaiC*[23]. Previous proteomic analysis showed that one of the key circadian clock proteins, KaiB (Slr0757), was induced [9]. RNA-Seq transcriptomic analysis showed that *kaiC* gene (*slr0758*) was also induced (Table 3). Transcripomics analysis here complemented well with the proteomic analysis, further confirming that circadian rhythms are induced by ethanol treatment. The ethanol-induced genes were listed in Table 3, while the induced genes encoding hypothetical proteins were provided in Additional file 3: Table S3.

Cross-membrane transporters for small molecules have been suggested as one important mechanism against ethanol toxicity in the early studies with yeast [24,25]. In cyanobacteria, transporters were also involved in tolerance to many different types of stresses, such as arsenate, Cu^2+^, salinity and heavy metals [26-30]. Our quantitative proteomic analysis also identified 5 putative transporters with different substrate specificity induced by ethanol exposure [9]. RNA-Seq based transcriptomics found 12 transporters were induced by ethanol at varying growth phases. Similarly, these transport proteins were also with a wide range of putative functions and substrate specificity: *sll0759* encoding an ABC transporter ATP-binding protein, *slr0949* encoding an integral membrane protein of the ABC-type Nat permease, *sll0540* encoding a phosphate-binding protein PstS homolog, *sll0671* encoding a probable cation transporter, *sll0536* encoding a probable potassium channel protein, *sll1428* encoding a probable sodium-dependent transporter, *slr2131* encoding a RND multidrug efflux transporter, *sll0384* encoding a cation and iron carrying protein, *sll1041* encoding a sulfate transport ATP-binding protein CysA, *sll0374* encoding a urea transport system ATP-binding protein, and *slr0678* encoding a biopolymer transport ExbD like protein, and *slr1452* encoding a sulfate transport system substrate-binding protein. Interestingly, they represented a totally different set of ethanol-induced transporters when compared with transporters revealed by proteomics analysis [9], although they shared some similarity in terms of substrate specificity as two of previously identified transporters, Sll0689 as a sodium-dependent transporter and Slr1295 as an iron transporter.

Early studies have found that many microbes can modify their cell membrane and envelope to increase tolerance to ethanol [24,31]. One well described change is the shift from *cis* to *trans* unsaturated fatty acids to decrease membrane fluidity, resulting in a corresponding increase in solvent tolerance [8]. RNA-seq transcriptomics analysis showed that *slr1350* encoding acyl-lipid desaturase was up-regulated at 72 h. In a previous study, the acyl-lipid desaturase (*desA*) gene from *Synechocystis* sp. PCC6803 was expressed in prokaryotic (*E. coli*) and eukaryotic (*Solanum tuberosum*) cells, which led to an enhanced cold tolerance due to increased unsaturated fatty acid concentration in their lipids [32]. Several genes encoding cell envelope proteins were found induced by ethanol exposure (Table 3). The *slr0819* gene encoding apolipoprotein N-acyltransferase was induced 2.17 and 1.67 fold in both biological replicates at 72 h. Apolipoprotein N-acyltransferase is able to transfer an acyl group from sn-1-glycerophospholipid to the free alpha-amino group of the N-terminal cysteine of apolipoproteins, resulting in mature triacylated lipoprotein which plays important role in bacterial survival in mice for *Staphylococcus aureus*[33,34]. The *sll1370* gene encoding a mannose-1-phosphate guanylyltransferase was induced 3.25 and 1.88 fold in both biological replicates at 72 h. Mannose-1-phosphate guanylyltransferase is involved in lipopolysaccharide biosynthesis which has been found necessary for adaptation to high external NaCl stress in *Rhizobium tropici*[35]. The *slr1910* gene encoding a probable N-acetylmuramoyl-L-alanine amidase was induced 1.71 and 2.00 fold in both biological replicates at 72 h. N-acetylmuramoyl-L-alanine amidase has been suggested involved in degradation and reconstruction of the cell peptidoglycan layer in *Anabaena* sp. strain PCC 7120 [36]. Up-regulation of these cell envelope proteins by ethanol exposure could contribute to strengthening cell wall and extracellular matrix for stress resistance, although the mechanism still needs more investigation.

Polyhydroxyalkanoates (PHAs) are highly reduced bacterial storage compounds that are accumulated in most bacteria during unbalanced growth conditions [37]. Accumulation and degradation of PHAs endow bacteria with enhanced survival, competition abilities, and stress tolerance, increasing fitness in changing environments [37,38]. RNA-seq analysis identified two genes involved in PHA biosynthesis, *slr1994* encoding a PHA-specific acetoacetyl-CoA reductase and *slr1993* encoding a PHA-specific beta-ketothiolase were up-regulated. Genetic analysis suggested that these two genes were probably located in the same operon [39]. Among them, *slr1994* encoding PHA-specific acetoacetyl-CoA reductase was up-regulated significantly at all three time points (*i.e.* 24, 48 and 72 h) with 6.0 and 9.0 fold increase in both biological replicates at 24 h (Table 3). Although PHA accumulation has been reported for many natural stress conditions [38], it is the first time to report that this pathway is also responsive to organic solvents and biofuels.

One factor that may affect the long-term survival of bacterial cells in a population is the level of damage incurred by macromolecules via the nonenzymatic process of glycation, which is responsible for the formation of several compounds identified as advanced glycation end products (AGEs) [40]. Many biochemical pathways produce reactive dicarbonyl intermediates, such as glyoxal and methylglyoxal (MG), which can further react with DNA, proteins, or other biomolecules to form AGEs [40]. In *E. coli*, it has been found that the predominant MG detoxification system consisted of glyoxalase enzyme I which coverts MG to S-lactoyl glutathione [41]. In plant, the level of MG is enhanced upon exposure to different abiotic stresses and overexpression of glyoxalase pathway genes can support survival and growth of transgenic plants under various abiotic stresses [42]. RNA-Seq analysis of the ethanol-treated cells showed that lactoylglutathione lyase (also called as glyoxalase enzyme I) was up-regulated significantly by 5.14 and 5.0 fold in both biological replicates at 72 h, suggesting that glyoxalase pathway may play important roles in resistance to ethanol stress in *Synechocystis*.

In the previous proteomics analysis, we unexpectedly discovered that many proteins involved in multiple aspects of photosynthesis activity (*i.e.* photosystem I and II, cytochrome, ferredoxin) were up-regulated even when the cell growth was slow down. We further confirmed the results by comparatively measuring chlorophyll a concentration in cells [9]. Based on our results we proposed that ethanol treatment might enhance photosynthesis in *Synechocystis* to generate more ROS which will trigger oxidative stress response [9]. RNA-seq transcriptomics analysis showed very similar results, although cell growth was slow, and genes involved in energy metabolism and protein synthesis were mostly down-regulated (Figure 2 and Additional file 2: Table S2), the genes involved in photosystem I and II, light collection and electron transfer, such as *ssl0563* encoding photosystem I subunit VII, *smr0009* encoding photosystem II PsbN protein, *sll1051* encoding phycocyanin alpha-subunit phycocyanobilin lyase, and *sll1471* encoding a phycobilisome rod-core linker polypeptide, and *slr1828* encoding a ferredoxin were up-regulated. Among them, *sll1051* encoding phycocyanin alpha-subunit phycocyanobilin lyase was increased significantly by 8.0 and 13.0 folds in both biological replicates at 72 h (Table 3). In addition, up-regulation of multiple cytochromes, such as *slr1185* encoding cytochrome *b6-f* complex alternative iron-sulfur subunit, *sll1316* encoding cytochrome *b6-f* complex iron-sulfur subunit, *sll0450* encoding cytochrome *b* subunit of nitric oxide reductase, *smr0003* encoding cytochrome *b6-f* complex subunit PetM were also up-regulated. The results further confirmed this unique phenomenon of cyanobacteria under stress of biofuels.

RNA-seq transcriptomics analysis identified ten signal transduction proteins induced upon ethanol exposure, including two histidine kinases (*sll1473*, *slr1805*), two response regulators (*slr0947*, *sll1330*) of bacterial two-component system (TCS), one serine/threonine kinase (*slr1225*) and three transcriptional regulators (*slr0741*, *sll0792*, *sll1423*, *ssl0707*) (Table 3). *sll1473* encoding a phytochrome-like sensor histidine kinase, was up-regulated at 48 h. Phytochromes are red/far-red photoreceptors that bear linear tetrapyrrole (bilin) chromophores attached to an N-terminal sensory module, and have been identified in many prokaryotes, including cyanobacteria [43,44]. In a study, the *cikA* gene of the cyanobacterium *Synechococcus elongatus* PCC 7942, encoding a phytochrome-related histidine kinase, was found involved in signal perception for resetting the circadian clock in response to environmental cues [45]. Although still needs more proof, the up-regulation of *sll1473* gene may be consistent with the enhanced expression of *kaiC* gene (*slr0758*) related to circadian rhythms. *slr0947* encoding a response regulator for energy transfer from phycobilisomes to photosystems was up-regulated at 72 h after ethanol exposure. Early study has found that RpaB response regulator (Slr0947) can bind to the upstream region of the high light (HL)-inducible genes in *Synechocystis* sp. PCC 6803 to cope with the potentially damaging effects of high light [46]. *slr0741* encoding transcriptional regulator was up-regulated at 72 h. The gene was previously found involved in transduction of the phosphate-limitation signal in *Synechocystis*[47]. *sll1330* encoding a two-component system response regulator OmpR subfamily was induced by ethanol at 48 h. A recent study found that expression of *sll1330* can be enhanced by nitrogen depletion under the control of NtcA, which then activates transcript accumulation of sugar catabolic genes during nitrogen starvation [48]. *slr1805* encoding a two-component sensor histidine kinase was up-regulated, which was previous found participating in the perception and transduction of salt-stress and hyperosmotic-stress signals [49].

**Figure 4:**
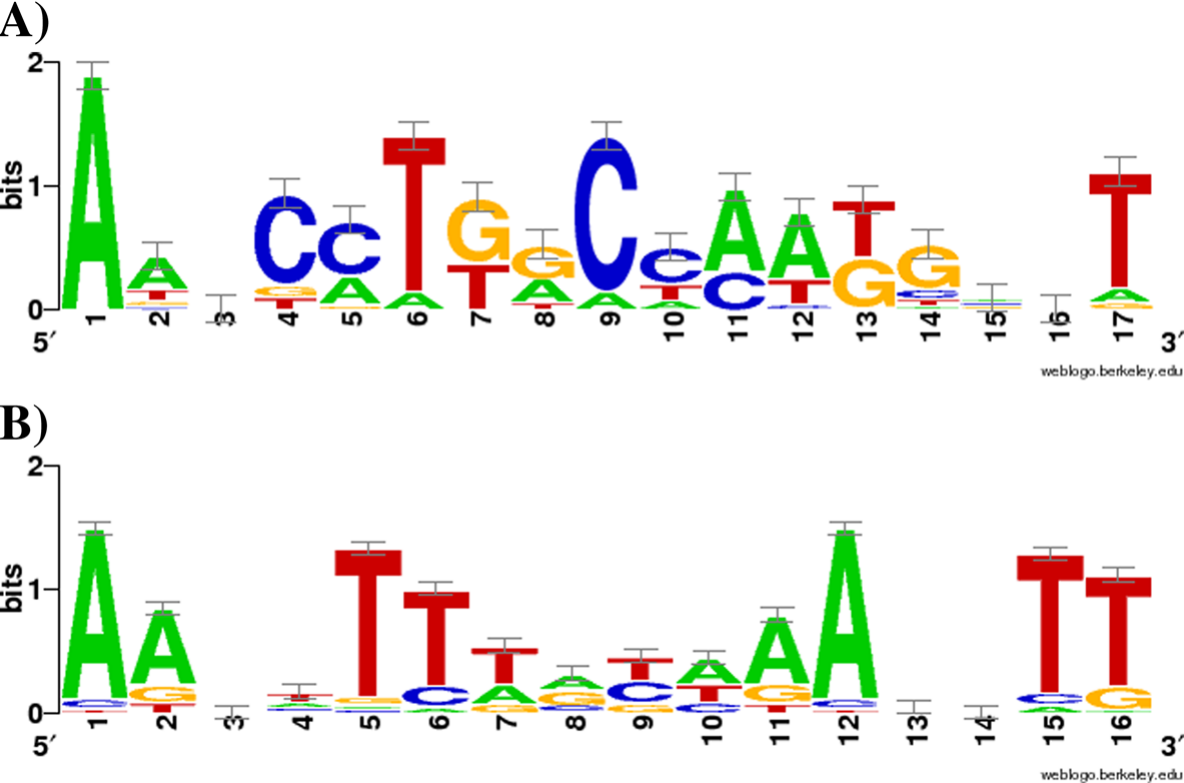
**Putative regulatory module identified upstream of common-responsive genes.** The motif is represented by a sequence logo generated by the WebLogos software [52].

Considering many signal transduction genes were involved in the ethanol induced responses, we speculated that some of the ethanol-responsive genes may be under direct control of the response regulators or transcriptional regulators. To seek evidence to this hypothesis, we performed a promoter DNA-binding motif searching using 500 bp sequences extracted from upstream region of all the up-regulated genes using the Gibbs Motif Sampler software [50,51]. This analysis showed that the top conversed motifs identified were two palindrome containing 16 and 17 total sites with the DNA sequence “AXXCCTGGCCAAGGXXT” and “AAXXTTTXXAAAXXTT”, respectively (Figure 4) [52]. Both motif models have several conserved positions with information bits greater than 0.5 and are highly likely to be significant [50]. The genes associated with the first motif included *slr0086* encoding a DnaK protein and *slr0942* encoding an alcohol dehydrogenase [NADP^+^ which have been confirmed in ethanol resistance in *Clostridium*[53] and *sll1330* encoding a OmpR subfamily response regulator which was shown Sll1330 to control the expression of glycolytic genes in *Synechocystis* sp. PCC 6803 [54]. The genes associated with the second motif included *slr1109* encoding an ankyrin, *slr1828* encoding a ferredoxin, *slr1994* encoding a PHA-specific acetoacetyl-CoA reductase, *slr2033* encoding a membrane-associated rubredoxin and *slr0940* encoding a zeta-carotene desaturase, which were all involved in stress response in various microbes. Functions of these motifs may worth further investigation.

### Correlation of transcriptomic and proteomic analyses

**Figure 5:**
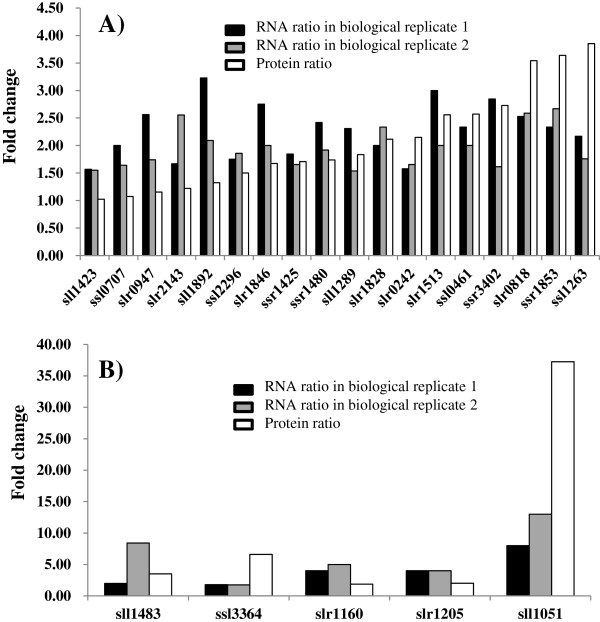
**Comparison of ratios derived from RNA-Seq based transcriptomics with those from proteomics for up-regulated genes/proteins by ethanol. A**) Responsive genes/proteins with fold change smaller than 5.0; **B**) Responsive genes/proteins with fold change greater than 5.0.

While it is well-known that RNA expression and protein abundance are not always correlated well [55,56], we have presented evidences above that overall cellular responses identified from transcripomics and proteomics are very similar: responses such as induction of common stress response, transporters, cell envelope proteins and photosynthesis were observed in both proteomic and transcriptomic datasets. To further compare the proteomic and transcriptomic datasets quantitatively, twenty-three common genes/proteins up-regulated in both transcriptomics and proteomics datasets were plotted together (Figure 5). The results also showed very similar trends of up-regulation, with only five genes up-regulated in transcriptomic data, but almost no change in proteomics dataset (*i.e*. *sll1423*, *ssl0707*, *slr0947*, *slr2143* and *sll1892*). However, no gene/protein with opposite regulation direction was found. In *Saccharomyces cerevisiae*, it has been proposed that there are three potential reasons for the lack of a strong correlation between transcriptomic and proteomic datasets: *i*) translational regulation, *ii*) difference in protein half-lives *in vivo* and *iii*) significant levels of experimental error, including differences with respect to the experimental conditions being compared [57,58]. The inconsistence between transcriptomic and proteomic datasets also highlighted that it may not be enough to analyze biological systems only at a single level.

### Validation of the potential resistance targets by mutant strains

**Figure 6:**
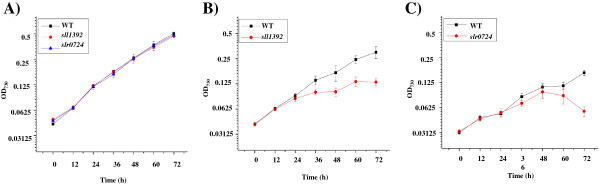
**Ethanol tolerance analysis of mutant strains. A**) Growth time courses of wild type, *sll1392* and *slr0724* mutants in BG11 medium; **B**) Growth time courses of wild type and *sll1392* mutant in BG11 supplemented with 1.5% ethanol; **C**) Growth time courses of wild type and *slr0724* mutant in BG11 supplemented with 1.5% ethanol.

Two genes, *slr0724* and *sll1392* which were found induced by ethanol exposure at 72 h for 1.5-2.0 and 4.0-5.0 folds, respectively (Table 3, Additional file 3: Table S3), were selected for construction of knockout mutants and for validation of their involvement in ethanol resistance. *slr0724* encodes a HtaR suppressor protein homolog (*sohA*, or *prlF*) according to CYORF Cyanobacteria Gene Annotation Database ( http://cyano.genome.ad.jp/), and *sll1392* encodes a regulatory gene, designated as *pfsR* (photosynthesis, Fe homeostasis and stress-response regulator) [59]. After confirmation by PCR and sequencing analysis, the mutants were grown in parallel with wild type *Synechocystis* sp. PCC 6803 in both normal BG11 medium and the BG11 medium supplemented with 1.5% ethanol. Comparative analysis showed that although there is no visible difference in terms of growth patterns between the wild type and the mutants in BG11 medium (Figure 6A), the *slr0724* and *sll1392* mutants grew slower than the wild type under 1.5% ethanol (Figure 6BC), suggesting that the mutants are more sensitive to ethanol, and the gene *slr0724* and *sll1392* may be involved in ethanol resistance. In addition, the results also showed that the growth difference between the wild type and the mutants became more significant at the late growth phases (*i.e.* 60–72 h), consistent the transcriptomic results that both genes were up-regulated only at 72 h (Table 3, Additional file 3: Table S3). According to NCBI annotation (NCBI accession ID: NP_439991.1), the *slr0724* gene could be involved in protein secretion and it induces growth defect when overproduced or mutated; however, under our growth condition, no difference in terms of growth was observed between the mutant and the wild type strains (Figure 6A). In addition, the PrlF mutation was found to induce the activity of the Lon protease. In prokaryotic cells the ATP-dependent proteases Lon are involved in the turnover of misfolded proteins and the degradation of regulatory proteins, and depending on the organism, these proteases contribute variably to stress tolerance [60,61]. Early studies have shown that *lon* mutants of *Campylobacter jejuni* grow poorly at high temperature [60] and Lon protease is involved in the control of the SOS response, acid tolerance and nutritional deprivation in *Escherichia coli*[61]. It still needs more proof whether the similar biological process was also functional in PCC6803 against ethanol. An early study has found that the *sll1392* (*pfsR*) deletion mutants were less sensitive to iron limitation under low light conditions and to suffer less lipid peroxidation following exposure to high light, suggesting a critical role of PfsR in regulation of iron homeostasis and stress response [59]. It may worth further investigation of the relationship between ethanol stress and iron homeostasis in *Synechocystis* sp. PCC 6803.

## Conclusions

To fully elucidate microbial metabolism and its responses to ethanol, it is necessary to include functional characterization and accurate quantification of all levels of gene products, mRNA, proteins and even metabolites [54]. While high-throughput ‘omics’ approaches to analyze molecules at different cellular levels are rapidly becoming available, it is also becoming clear that any single ‘omics’ approach may not be sufficient to characterize the complexity of biological systems. To provide confirmation to previous proteomic analysis and also to reveal more responses at transcriptional level, in the study, we applied a quantitative RNA-Seq based transcriptomics approach combined with quantitative reverse-transcript PCR (RT-PCR) analysis to reveal the global transcriptomic responses to ethanol in *Synechocystis* sp. PCC 6803. The results showed that *Synechocystis* probably employed multiple and synergistic resistance mechanisms in dealing with ethanol stress. In addition, we found that the overall cellular responses inferred from transcriptomic and proteomic analyses were very similar, although the responsive genes were not always the same. By constructing knockout mutants and analyzing their ethanol tolerance, we have provided preliminary validation that the targets identified by the study could be used to obtain ethanol-tolerant cyanobacterial hosts by genetic engineering in *Synechocystis* sp. PCC 6803. Finally, our results showed that gene knockout of the potential targets individually caused only partial loss of the ethanol tolerance, consistent with the early conclusion that microbes tend to employ multiple resistance mechanisms in dealing with stress of single biofuel product [7,8]. With the ethanol-tolerance gene targets discovered from this study and previously proteomic analysis [9], it may be possible to engineer multiple gene targets from different cellular functional categories simultaneously to achieve high-tolerance hosts in the future.

## Methods

### Bacterial growth conditions and ethanol treatment

*Synechocystis* sp. PCC 6803 was grown in BG11 medium (pH 7.5) under a light intensity of approximately 50 μmol photons m^-2^ s^-1^ in an illuminating incubator of 130 rpm at 30°C (HNY-211B Illuminating Shaker, Honour, China). Cell density was measured on a UV-1750 spectrophotometer (Shimadzu, Japan). For growth and ethanol treatment, 10 mL fresh cells at OD_730_ of 0.5 collected by centrifugation and then were inoculated into 50 mL BG11 liquid medium in a 250-mL flask. Ethanol of varying concentration was added at the beginning of cultivation. 1 mL of culture samples were took and measured (OD730) every 12 h. Morphology of *Synechocystis* sp. PCC6803 control and ethanol-treated samples was observed using a BX43 fluorescence microscope (Olympus, Japan). Cells for transcriptomics analysis were collected by centrifugation at 8,000 x *g* for 10 min at 4°C.

### RNA preparation and cDNA synthesis

Approximately 10 mg of cell pellets were frozen by liquid nitrogen immediately after centrifugation and cell walls were broken with mechanical cracking at low temperature. Cell pellets were then resuspended in Trizol reagent (Ambion, Austin, TX) and mixed well by vortex. Total RNA extraction was achieved using a miRNeasy Mini Kit (Qiagen, Valencia, CA). Contaminating DNA in RNA samples was removed with DNase I according to the instruction in the miRNeasy Mini Manual (Qiagen, Valencia, CA). The RNA quality and quantity were determined using Agilent 2100 Bioanalyzer (Agilent, Santa Clara, CA) and subjected to cDNA synthesis. The RNA integrity number (RIN) of every RNA sample used for sequencing was more than 8.0. For each sample, 500 ng total RNA were subjected to cDNA synthesis using a NuGEN Ovation® Prokaryotic RNA-Seq System according to manufacturer's protocol (NuGEN, San Carlos, CA). The resulting double-stranded cDNA was purified using the MinElute Reaction Cleanup Kit (Qiagen, Valencia, CA).

### RNA-seq library preparation

The double-stranded cDNA obtained was subjected to library preparation using the Illumina TruSeqTM RNA Sample Preparation Kit (Illumina, San Diego, CA), through a four-step protocol of end repairing, adding adenylate 3’ ends, adapter ligation, and cDNA template enrichment. Amplification program is: 98°C 30 s; 98°C 10 s, 60°C 30 s, 72°C 30 s for 15 cycles; 72°C for 5min, and then hold at 4°C. To determine the quality of the libraries, a Qubit® 2.0 Fluorometer and Qubit™ dsDNA HS (Invitrogen, Grand Island, NY) were first used to determine the DNA concentration of the libraries, and then FlashGel DNA Cassette (Lonza, USA) or Agilent Technologies 2100 Bioanalyzer (Agilent, Santa Clara, CA) was used to determine the product size of the libraries, with good libraries typically around 300 bp. The product was used directly for cluster generation using Illumina's Solexa Sequencer according to the manufacturer's instructions.

### Next-generation sequencing

RNA 2×100 bp paired-end sequencing was performed using Illumina’s Solexa Genome Analyzer II using the standard protocol. The cDNA library of each sample was loaded to a single lane of an Illumina flow cell. The image deconvolution and calculation of quality value were performed using Goat module (Firecrest *v*.1.4.0 and Bustard *v*.1.4.0 programs) of Illumina pipeline *v*.1.4. Sequenced reads were generated by base calling using the Illumina standard pipeline.

### Transcriptomics data analysis

Sequence reads were pre-processed using FASTX Toolkit (Version: 0.0.13) to remove low-quality bases, and reads shorter than 20 bp. The qualified sequence reads were then mapped to non-coding RNA (*nc*RNA) sequences using Bowtie (Version: 2.0.0) with default settings. Genome sequences (including *nc*RNA sequences) and annotation information of *Synechocystis* sp. PCC 6803 were downloaded from NCBI and the Comprehensive Microbial Resource (CMR) of TIGR ( http://www.tigr.org/CMR) (Downloaded on April 22, 2012) [10]. Reads that mapped to *nc*RNA sequences were excluded from further analysis. For paired-end Illumina reads, both pairs were removed if either pair mapped to rRNA. Remaining reads were mapped to the *Synechocystis* sp. PCC 6803 genome using Bowtie (Version: 2.0.0) with the default parameters. For gene expression determination, we performed a standard calculation of Reads Per Kilobase of Gene Per Million Mapped Reads (RPKM) based on the following formula [13]:

$$\begin{array}{c} \mathrm{RPKM}=\\ \frac{\mathrm{transcription}\_\mathrm{reads}}{\mathrm{transcription}\_\mathrm{length}\;X\;\mathrm{total}\_\mathrm{assembly}\_\mathrm{reads}\_\mathrm{in}\_\mathrm{run}}\\ \;\times 10^{9} \end{array}$$

in which “transcription_reads” stands for the number of reads mapped to a given gene; transcription_length stands for gene length; and “total_mapped_reads_in_run” stands for the total number of reads in a given measurement. For each time point, two biological replicates of ethanol-treated samples and their control were analyzed and the corresponding gene expression ratios based on RPKM were calculated, the genes with 1.5 fold changes in both biological replicates were determined as differentially regulated genes.

### Quantitative real-time RT-PCR analysis

The RNA samples were collected from cells grown under the same growth condition as described above for transcriptomic analysis. Approximately 10 mg of cell pellets were frozen by liquid nitrogen immediately after centrifugation and cell walls were broken with mechanical cracking at low temperature. Cell pellets were then resuspended in Trizol reagent (Ambion, Austin, TX) and mixed well by vortex. Total RNA extraction was achieved using a miRNeasy Mini Kit (Qiagen, Valencia, CA). First-strand cDNAs were synthesized using RevertAidTM Reverse Transcriptase (Fermentas, Glen Burnie, MD). cDNA was subjected to eight hundred fold dilutions, and 2 μl of each dilution was used as template for following qPCR reaction. The qPCR reaction was carried out in 20 μl reactions containing 10 μl of SYBR® Green PCR Master Mix (Applied Biosystems, Foster City, CA), and 2 μl of each PCR primer at 2 mM, employing the StepOnePlus™ Real-Time PCR System (Applied Biosystems, Foster City, CA), under the following condition: 50°C for 2 min and 95°C for 10 min, followed by 40 cycles of 95°C for 15 s and 60°C for 1 min. Quantification of gene expression was determined according to standard process of RT-PCR which used serial dilutions of known concentration of chromosome DNA as template to make a standard curve. A total of 18 selected genes based on their differential expression patterns revealed by iTRAQ were selected for verification and the *rnpB* gene (*6803s01*) encoding RNase P subunit B was used as an internal control according to the previous publication [62]. Three technical replicates were performed for each gene. Data analysis was carried out using the StepOnePlus analytical software (Applied Biosystems, Foster City, CA). Briefly, the amount of relative gene transcript was normalized by that of *rnpB* in each sample (wild type or mutant), using the following method:

$$\begin{array}{cc} R_{\mathrm{relative}\;\mathrm{gene}\;\mathrm{expression}\;\mathrm{of}\;\mathrm{gene}x} & =2^{\left({\mathrm{Ct}\;}_{\mathrm{control}}–{\mathrm{Ct}\;}_{\mathrm{treated}}\right)\;\mathrm{of}\;x}\\ & \;/2^{\left({\mathrm{Ct}\;}_{\mathrm{control}}-{\mathrm{Ct}}_{\mathrm{treated}}\right)\;\mathrm{of}\;\mathrm{rnpB}} \end{array}$$

Then data was presented as ratios of the amount of normalized transcript in the treatment to that from the control. The gene ID and their related primer sequences used for real-time RT-PCR analysis were listed in Additional file 4: Table S4.

### Promoter analysis and motif identification

The Gibbs Motif Sampler software from the Biometrics Laboratory of Wadsworth Center, ( http://www.bayesweb.wadsworth.org/gibbs/gibbs.html), was used to identify matrix models describing DNA sequence motifs present upstream of genes responsive to ethanol treatment [50,51]. Regions representing approximately 500 base pairs of the DNA sequences upstream of the translational start site of genes responsive to ethanol stress were extracted from the NCBI genome database using the Regulatory Sequence Analysis Tools (RSAT) [63]. Both strands of each sequence were searched and possible motif locations were identified using the motif matrix score obtained from the Gibbs Motif Sampler software. The multilevel consensus sequence for each motif was then used to generate a sequence logo that is a graphical representation of nucleic acid multiple sequence alignment ( http://www.weblogo.berkeley.edu/) [52].

### Construction and analysis of gene knockout mutants

A fusion PCR based method was employed for the construction of gene knockout fragments [64]. Briefly, for the gene target selected, three sets of primers were designed to amplify a linear DNA fragment containing the chloramphenicol resistance cassette (amplified from a plasmid pACYC184) with two flanking arms of DNA upstream and downstream of the targeted gene. The linear fused PCR amplicon was used directly for transformation into *Synechocystis* sp. PCC 6803 by natural transformation. The chloramphenicol-resistant transformants were obtained and passed several times on fresh BG11 plates supplemented with 10 μg/ml chloramphenicol to achieve complete chromosome segregation (confirmed by PCR). Two genes, *slr0724* and *sll1392* that have been found differentially regulated by ethanol exposure, were selected for construction of gene knockout mutants. The mutants were confirmed by PCR and sequencing analysis. PCR primers for mutant construction and validation were listed in Additional file 4: Table S4. Comparative growth analysis of the wild type 6803 and the mutants were performed in 100-mL flasks each with 10 mL BG11 medium with or without 1.5% ethanol. Cultivation conditions are the same as described above. Growth analysis was performed in triplicates.

## Abbreviations

*Adh*: Alcohol dehydrogenase; AGES: Advanced glycation end products; CMR: Comprehensive Microbial Resource; *desA*: Acyl-lipid desaturase; HL: High light; iTRAQ: Isobaric tag for relative and absolute quantitation; LC-MS/MS: Liquid chromatography-tandem mass spectrometry; MG: Methylglyoxal; ncRNA: non-coding RNA; PHA: Polyhydroxyalkanoate; *pdc*: Pyruvate decarboxylase; PSII: Photosystem II; RPKM: Reads per kilobase of gene per million mapped reads; ROS: Reactive oxygen species; RSAT: Regulatory sequence analysis tools; RT-PCR: Reverse-transcript PCR; TCS: Two-component system.

## Competing interest

The authors declare that they have no competing interests.

## Authors’ contributions

JW, LC and WZ conceived of the study. JW, LC, JQ and WZ drafted the manuscript. JW, LC, and XT carried out cultivation and transcriptomics analysis. LC, JL, XR and SH carried out the RT-PCR, mutant construction and phenotypic analysis. SH finished the promoter analysis. JW, LC and WZ finish the statistical analysis for transcripomic data. All authors read and approved the final manuscript.

## Supplementary Material

Additional file 1 **Table S1. **Raw RNA-seq transcriptomics data. Click here for file

Additional file 2 **Table S2. **Gene down-regulated by ethanol exposure. Click here for file

Additional file 3 **Table S3. **Induced genes encoding hypothetical proteins. Click here for file

Additional file 4 **Table S4. **Primers used in this study. Click here for file
